# Genome-Scale Metabolic Modeling of Archaea Lends Insight into Diversity of Metabolic Function

**DOI:** 10.1155/2017/9763848

**Published:** 2017-01-04

**Authors:** ShengShee Thor, Joseph R. Peterson, Zaida Luthey-Schulten

**Affiliations:** ^1^Center for Biophysics and Quantitative Biology, University of Illinois at Urbana-Champaign, 600 South Mathews Avenue, Urbana, IL 61801, USA; ^2^Department of Chemistry, University of Illinois at Urbana-Champaign, 600 South Mathews Avenue, Urbana, IL 61801, USA; ^3^Carl R. Woese Institute for Genomic Biology, 1206 W Gregory Dr., Urbana, IL 61801, USA

## Abstract

Decades of biochemical, bioinformatic, and sequencing data are currently being systematically compiled into genome-scale metabolic reconstructions (GEMs). Such reconstructions are knowledge-bases useful for engineering, modeling, and comparative analysis. Here we review the fifteen GEMs of archaeal species that have been constructed to date. They represent primarily members of the Euryarchaeota with three-quarters comprising representative of methanogens. Unlike other reviews on GEMs, we specially focus on archaea. We briefly review the GEM construction process and the genealogy of the archaeal models. The major insights gained during the construction of these models are then reviewed with specific focus on novel metabolic pathway predictions and growth characteristics. Metabolic pathway usage is discussed in the context of the composition of each organism's biomass and their specific energy and growth requirements. We show how the metabolic models can be used to study the evolution of metabolism in archaea. Conservation of particular metabolic pathways can be studied by comparing reactions using the genes associated with their enzymes. This demonstrates the utility of GEMs to evolutionary studies, far beyond their original purpose of metabolic modeling; however, much needs to be done before archaeal models are as extensively complete as those for bacteria.

## 1. Introduction

Since their discovery and classification in the late 1970s and early 1980s [1–5] archaea have garnered considerable interest, due in part to prevailing thoughts at the time that they lived primarily in extreme conditions, a property that results in unique cell physiology and metabolic characteristics [6]. Although the original classification of organisms was based on only thirteen sequences with only four representatives of archaea [2], the proposal of the three domains of life has been tested time and time again [6–10] and holds up well. Archaea have now been found to reside in essentially every terrestrial environment, and the unique natural capability of methane production among certain archaeal groups makes this domain of life remarkably novel.

Despite the significant progress in sequencing archaeal genomes, a systematic understanding of the metabolism of archaea is still lacking. This is especially true for peripheral metabolic pathways and mechanisms of adaptation to extreme environments [11]. It has often been noted that the environmental niches dominated by archaea constitute extremely stressful or even fatal homes for their bacterial cousins; thus, they have evolved unique coping mechanisms and optimized their metabolisms to salvage the energy that would otherwise be left unused in the environment. It has been proposed that adaptation to energy stress could be the primary factor driving the evolution of archaea [12]. The consequence would be that they have evolved specialized tolerance and metabolic capabilities unique to their environments which make them relatively inflexible to adaptation like their bacterial counterparts. It has been proposed that this inflexibility possibly results in tighter phylogenetic groups that directly represent less metabolic diversity [12]. Indeed, the evidence seems to support this hypothesis as only 89 genera of archaea have been identified in contrast to the over 1,400 bacterial genera. This fact should be exploitable by systems biology researchers as it means that information gained by one member of a taxon can largely be extended to other related members of the taxa.

**Figure 1:**
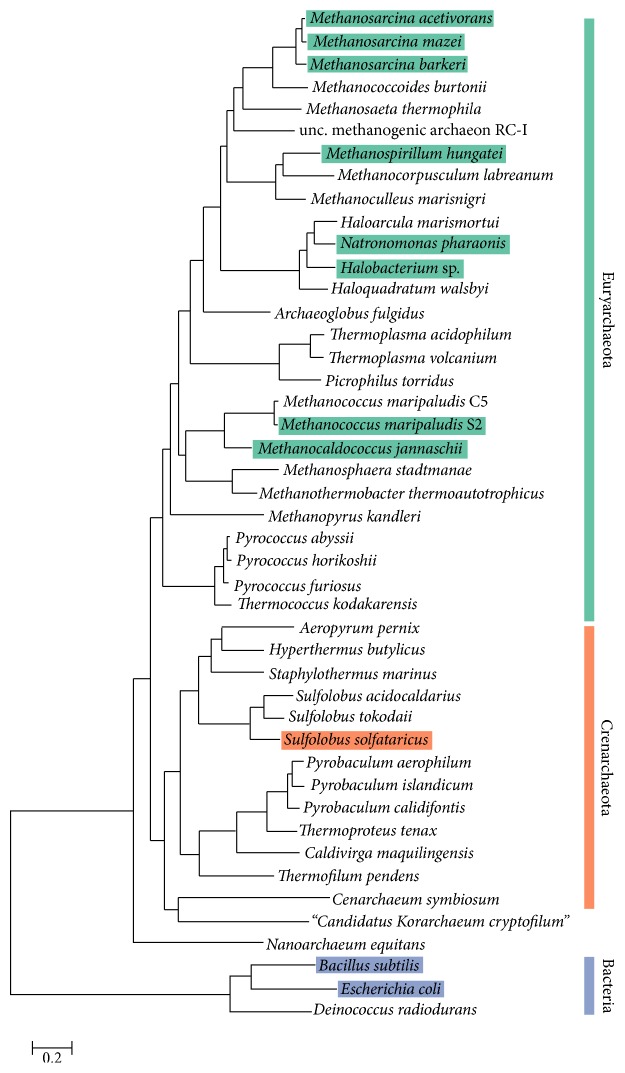
Diversity of archaeal models. A visualization of the diversity of archaeal genome-scale metabolic models as related by phylogeny. The figure is adapted from Elkins et al. [27] where the maximum-likelihood tree was constructed using 33 conserved ribosomal proteins and three largest RNA polymerase subunits. Highlighted species indicate that genome-scale metabolic models have been constructed for that organism. Although not shown in this adapted figure, a* Methanobrevibacter smithii* model within the Euryarchaeota has also been constructed. While numerous models have been constructed for Euryarchaeota, the Crenarchaeota are severely underrepresented.

For this reason, systematic databases of the metabolic properties of the archaea are highly desirable; the field of systems biology is uniquely positioned to provide useful insight into the diversity and evolution of metabolic capabilities. To date, fifteen genome-scale metabolic models (GEMs; one of the main products of systems biology research) have been constructed for ten archaeal species. However, these models represent primarily members of the Euryarchaeota with almost three-quarters representatives of methanogens. An examination of the phylogenetic tree demonstrates a lack of well-curated metabolic reconstructions in many of the archaeal taxa (see Figure 1).

Despite the limited representation, much can already be learned from the GEM “knowledge-bases”. Here, we review the GEMs constructed to date and the knowledge gleaned from them. We begin by briefly reviewing the construction process for GEMs and general predictions made by metabolic models. We also give a historical perspective of the construction of the archaeal GEMs. We then review the models and specific insights gained from model constructions, including novel metabolic enzymes/pathways. Finally, we demonstrate the utility of these metabolic models to the study of evolution of diversity in archaea. We do this by computing the conservation of reactions (based on genetic association of the enzymes) across the archaea and visualizing the extent of conservation on a comprehensive map of the metabolism of the methanogen* Methanosarcina acetivorans*.

## 2. Genome-Scale Metabolic Models (GEMs)

Metabolic networks are invaluable tools for qualitatively understanding an organism's metabolic behavior under given conditions and have a long history of use in biology. Systematic construction of metabolic models which couple metabolic networks with genetic associations, reactions that exchange metabolites with the environment, and the organism's biomass composition only began to take shape in the mid-1990s when Fleischmann et al. [46] fully sequenced the entire genome of the bacterium* Haemophilus influenzae* Rd. Through comparative genomics they showed that 68% of the known* E. coli* proteins had homologs in the* H. influenzae* Rd genome, enabling a hypothesis of which metabolic pathways exist in* H. influenzae* Rd. Since then, the pioneering work of Thiele and Palsson [47] has established genome-scale metabolic models (GEMs) as the standard computational tool with which to quantitatively study the metabolic behaviors of organisms. In 2010, a well-established workflow was published in an article detailing the best practices for the model construction process [47].

A GEM can best be described as a knowledge-base containing all the biochemical information describing an organism's metabolic network. They are typically presented as Systems Biology Markup Language (SBML) [48] files that can be queried to obtain information about individual reactions, metabolites, and genes coding for the enzymes that catalyze the reactions. Software such as MATLAB COBRA Toolbox [49, 50] or COBRApy [51] that implement Constraint-Based Reconstruction and Analysis (COBRA) methods can then use the information within a GEM to compute predicted metabolic behaviors of the organism subject to specified environmental and physiological limitations [52]. Alternatively, one can create independent analysis tools that simply use GEMs to identify product synthesis pathways [53–56], optimize bioprocessing efficiency [57, 58], predict metabolic engineering targets [58, 59], and elucidate more complex phenomena such as symbiosis in microbial communities [24, 60–63].

### 2.1. Model Construction and Predictions

Here, we will briefly summarize the GEM construction process and highlight the most important characteristics of GEMs that one typically encounters. The de facto standard GEM construction protocol is that published by Thiele and Palsson [47] and should be referred to for standards within the field.

The construction process is divided into four broad stages: (1) automated construction of a draft model, (2) manual refinement of the draft model, (3) conversion of the model into a mathematical model, and (4) quantitative evaluation and refinement of the model. The first stage involves identifying all the potential reactions and pathways that the organism harbors based on its annotated genome. This process can be automated as it is essentially a bioinformatics problem requiring the comparison of the genome with databases that document known genes and their associated metabolic enzymes and pathways (e.g., KEGG [64], Uniprot [65], and BioCyc [66]). Many tools have been designed to facilitate this process such as the RAVEN toolbox [67], KBASE, PathwayTools [68], and the ModelSEED [69].

The manual curation stage of draft model refinement is the most time-consuming—arguably the most critical—portion of the process. All the reactions and pathways identified in the first stage are evaluated to ensure a variety of consistencies: experimental data should support their existence in the organism, masses and charges need to be balanced and consistent with reaction stoichiometries, and reaction directionalities need to be consistent with thermodynamic data. Missing pathways are added at this stage along with transport reactions responsible for the organism's influx and efflux of metabolites from the environment. The most crucial features that make GEMs unique and enable subsequent quantitative predictions are also established in this stage, specifically, the biomass objective reaction, the growth associated ATP maintenance reaction (GAM) and the corresponding nongrowth associated ATP maintenance (NGAM) reaction, and the Boolean gene-protein-reaction associations (GPRs).

Quantitative prediction using GEMs is typically framed as a linear programming problem in which one feature of the model is optimized under a given set of constraints. This feature is typically the model's biomass production rate (analogous to growth rate) which is described by a single pseudo-reaction that produces a “biomass” pseudo metabolite by consuming all the metabolites that the organism requires to grow (e.g., individual amino acids, carbohydrates, lipids, nucleic acids, vitamins, cofactors, ions, and trace metals). Ideally, this reaction is constructed using the experimentally characterized biomass composition of the organism. However, this data is often difficult to obtain, leaving curators to either estimate biomass compositions from the organism's genome or adopt the compositions available from other organisms.

The GAM and NGAM reactions consume ATP. The GAM reaction reflects the ATP consumption required for the organism to grow whereas the NGAM reaction reflects the organism's basal ATP consumption required to survive but not necessarily to grow (e.g., maintaining membrane potential and redox balance). Since both reactions reflect the organism's energy requirements in the model, the choice of stoichiometric coefficients for these two reactions greatly influences the model's growth predictions. Ideally, the stoichiometric coefficients of these two vital reactions should be determined from a chemostat experiment in which the growth rate is tracked alongside ATP consumption (or some fiducial metabolite tracing ATP consumption, e.g., as in [70]). In practice, one will find that researchers often use a variety of estimation schemes based on the experimental data at hand. The most common alternative method for determining ATP requirements involves matching the growth rate and growth yield (grams dry mass per substrate uptake or efflux) data from batch cultures grown in different media assuming a specific biomass composition and stoichiometric matrix (e.g., as in [19]). In absence of measurements for the NGAM, it is often assumed to be some fraction of the GAM (for example, 2.5% the GAM reaction in the 2006* M. barkeri* model by Feist et al. [19]).

The GPRs are Boolean expressions containing the genes that code for metabolic enzymes facilitating the reactions. By piecing the genes together in series of AND and OR operations, a GPR encodes which genes are necessary for an enzyme to be synthesized by the cell and therefore which genes are required for a metabolic reaction to exist. Predictions of gene knockout effects are commonly computed with GEMs. Not all reactions in the model will have GPRs due to either the lack of experimental gene characterizations, the use of nonphysical “gapfill” reactions [71], or the presence of novel uncharacterized pathways hypothesized by the curator.

Once this manual curation is complete, one can proceed to the third stage of converting the GEM into a quantitatively predictive model. This is done by defining the “objective” reaction to be optimized in the model and constraining the flux ranges on all model reactions. These flux ranges must reflect a specific growth condition to which the organism is subject. During model construction, most internal reaction fluxes will likely be unbounded, due to the relatively limited biochemical and proteomics data available for most reactions and organisms; it is the exchange reaction fluxes that must be constrained to reflect the nutrient availability of the organism's environment. Exchange reactions are nonphysical external reactions of the model that introduce compounds into the system, thereby simulating the organism's growth environment. These constraints [72] will have to be applied through COBRA-capable software. Once these model constraints have been set, flux balance analysis can be run to predict the organism's growth rate and the distribution of fluxes through the metabolic network. The fourth stage is validating these predictions with experimental growth data and discrepancies rectified with iterative manual refinement of the model. Numerous tools [67, 69, 73–78] have been developed over the years to automate many stages of this arduous construction process, allowing researchers to focus their effort on the last stage of model refinement. Dias et al. [73] provide a comparative review of these various computational tools.

## 3. Genealogy of Archaeal GEMs

**Figure 2:**
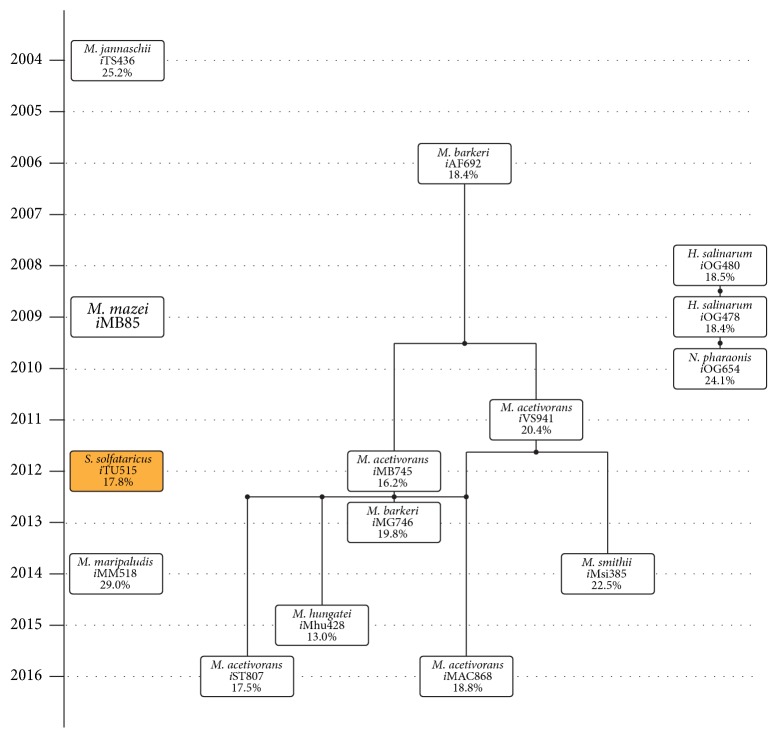
Genealogy of archaeal models. A diagram showing the evolution/genealogy of archaeal models since the reconstruction of* M. jannaschii* in 2004. Each box represents a single metabolic model and includes the species name, the name of the model, and the percentage of protein coding genes (where available) that are incorporated in the model. The sole representative of the Crenarchaeota kingdom [28] is highlighted in orange.

The genealogy of all the published archaeal GEMs to date is shown in Figure 2 (see Table 1 for statistics about the various models). The current archaeal GEMs can conveniently be divided between methanogenic and nonmethanogenic archaeal species with the former being the most developed due to the ecological roles that methanogens play in the global carbon cycle and their use in wastewater treatment [79]. Although the very first archaeal GEM was developed for* Methanococcus jannaschii* by Tsoka et al. [22] in 2003, the majority of the later methanogen GEMs were derived from a model for* Methanosarcina barkeri* (*i*AF692) which was first constructed by Feist et al. [19] in 2006. This inheritance stems from the fact that* i*AF692 was the first manually curated methanogen GEM thoroughly verified against experimental growth data.* M. barkeri* is also one of the most metabolically diverse methanogens in the Euryarchaeota kingdom, capable of consuming acetate, methylamines, methanol, CO, and CO_2_/H_2_. Two models,* i*VS941 [15] and* i*MB745 [16], were independently constructed for* M. acetivorans* and published in 2011 by the Maranas and Price research groups, respectively.* M. acetivorans* is equally diverse and similar in metabolism and thus inherited much of the* M. barkeri* GEM characteristics.* i*MB745 was then used as the base model from which to draft the more recent methanogen GEMs,* Methanobrevibacter smithii* (*i*Msi385) [24] and* Methanospirillum hungatei* (*i*Mhu428) [21], both of which were qualified as preliminary reconstruction for use in larger microbial community studies. The most recent* M. acetivorans* models include* i*MAC868 [17] and our own* i*ST807, which are both independent updates to* i*MB745. The GEM for* M. maripaludis* (*i*MM518) was constructed in 2014 by Goyal et al. [23, 80] independent of the other methanogen GEMs. This is not surprising given that by this time GEMs construction had already been well established.

Nonmethanogenic archaeal GEM construction has been largely dominated by the work from Dieter Oesterhelt's research group. In 2008, they released the first manually curated GEM for* Halobacterium salinarum* R-1 (*i*OG478) [13]. This was followed in 2010 by a new GEM for a haloalkaliphile,* Natronomonas pharaonis* (*i*OG654) [25], which inherited significantly from the* H. salinarum* model. The only other nonmethanogenic archaeal GEM to our knowledge was independently constructed in 2012 for the* Sulfolobus solfataricus* by Ulas et al. [26].

Although archaea had been established since the 1980s as the third domain of life by the pioneering work of Carl Woese and collaborators [3–5, 7, 10], the lack of experimental data on metabolic characteristics of various archaeal species explains why so few GEMs have been constructed to date. Nevertheless, this early stage of archaeal GEMs development provides a ripe opportunity for the community to grasp the core governing properties of archaeal metabolic networks and perhaps adopt standardized model building practices in order to facilitate more efficient communication of metabolic information among researchers going forward.

## 4. Methanogen GEMs

### 4.1. Methanogenesis Framework

**Figure 3:**
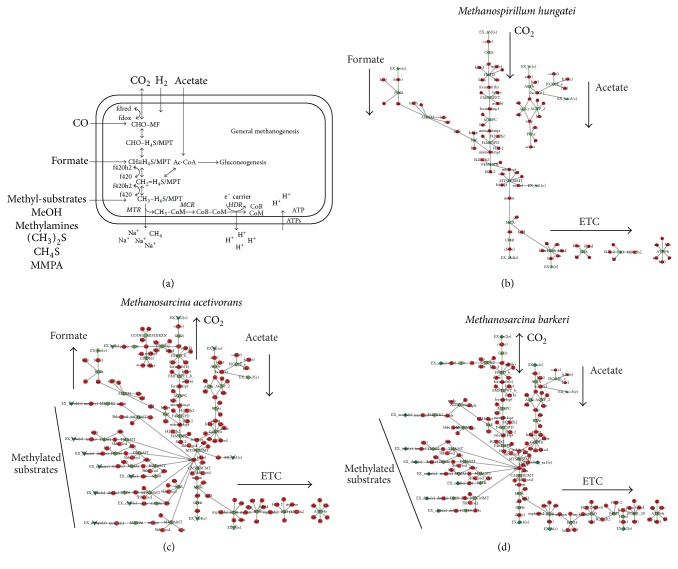
Methanogenesis pathway framework. (a) The basic structure of methanogenesis showing where the major growth substrates across all methanogens enter the pathway. The simplest methanogens are only capable of growing on CO_2_/H_2_ and/or formate while the most complex methanogens are also capable of methylotrophic and acetotrophic growth. H_4_S/MPT stands for tetrahydrosarcinapterin (H_4_SPT) and tetrahydromethanopterin (H_4_MPT). The former is found exclusively in Methanosarcinales whereas the latter is found in all other methanogens. (b) The hypothesized methanogenesis pathways for* M. hungatei* (*i*Mhu428) [21]. Although it cannot use acetate as an energy source, the pathway to take up acetate is still present to shuttle it into gluconeogenesis. (c) The hypothesized methanogenesis pathway for* M. acetivorans* (*i*ST807) [29]. The conventional CO_2_ reduction pathway is only run in reverse as this methanogen cannot metabolize CO_2_. (d) The hypothesized methanogenesis pathway for* M. barkeri* (*i*AF692) [19] which bears great resemblance to that of* M. acetivorans*. The major differences between the two organisms' methanogenesis pathways lie in the electron transport chain (ETC). The specific pathways for each methanogen follow the same topological structure as the general methanogenesis illustration. Red circles are metabolites while green diamonds signify enzymatic reactions of the pathway.

As the most defining metabolic pathway within methanogens, methanogenesis has been well characterized by numerous biochemical studies over the years. Therefore, the most significant and notable differences between the methanogen models will be found in the methanogenesis pathway and supportive pathways producing novel cofactors for different substrates. The basic framework is shown in Figure 3 where CO_2_ is reduced to methane in a series of steps. Although this basic framework is well conserved among methanogens, the key difference lies in the exergonic-endergonic reaction couplings in the pathway. The first step of CO_2_ reduction is an endergonic reaction that oxidizes ferredoxin and produces formylmethanofuran. In simple hydrogenotrophic methanogens that lack cytochromes, this energy is typically recovered by the methyl-H_4_MPT:CoM methyltransferase (Mtr) reaction and the heterodisulfide reductase (Hdr) reaction. Mtr expels Na^+^ ions in the process of transferring the methyl group onto coenzyme M (CoM) and thus establishes the electrochemical gradient responsible for driving ATP synthesis. Electrons are extracted from formate or H_2_ by Hdr which then uses these electrons to split the CoM-CoB heterodisulfide and reduce ferredoxin, thus replenishing the ferredoxin pool that is required to run the very first step of CO_2_ reduction. This oxidation of formate or H_2_ to reduce the heterodisulfide and ferredoxin is called the electron bifurcation reaction. This is in contrast to cytochrome-containing methanogens which are almost exclusively found within the Methanosarcinales. In these substrate-diverse methanogens, the Hdr enzyme evolved to harbor a cytochrome and can utilize methanophenazine as another electron carrier. Instead of directly reducing and replenishing the organism's supply of ferredoxin, Hdr expels hydrogen ions to establish a proton-based electrochemical gradient that is used by a membrane-bound energy conserving hydrogenase (Ech) to regenerate the reduced ferredoxin. This system is best exemplified by the* M. barkeri* model (*i*AF692) in which the Ech reaction was of particular interest during model construction because the ratio of protons translocated to electrons extracted was unknown at the time. Using experimental growth yield data, a stoichiometry of 1 proton/2e and GAM/NGAM of 70/1.75 mmol/gDWT/hr enabled the model to predict growth yields consistent with experimental data for growth on methanol, acetate, H_2_/CO_2_, and pyruvate. This Ech stoichiometry was later updated in* i*MG746 to 2 protons/2e^−^. Although very closely related to* M. barkeri*,* M. acetivorans* has significant differences as a marine methanogen. Within methanogenesis, it substitutes Ech with the ferredoxin:NAD^+^ oxidoreductase complex (Rnf) which interestingly translocates sodium ions instead of hydrogen ions [81]. This establishes a primarily Na^+^ dominated electrochemical gradient and helps explain why* M. acetivorans* inhabits a marine environment [40] in contrast to freshwater* M. barkeri* [82]. Since* M. acetivorans* is not able to consume CO_2_, it would not be carrying out the endergonic first step of reducing CO_2_ and thus justifies the absence of an Ech.

### 4.2. Methanogen GEMs

The majority of methanogenic GEMs available to date derive from the* M. barkeri* model* i*AF692 and the* M. acetivorans* model* i*MB745. Both models describe seven major metabolic subsystems: vitamins and cofactor biosynthesis, amino acid metabolism, nucleotide metabolism, central metabolism, lipid and cell wall biosynthesis, and methanogensis.* i*MB745 inherited most of the reactions in* i*AF692 but also incorporated various additional pathways. The most notable changes include a modification of the methanofuran biosynthesis pathway based on homology of enzymes to those from the same pathway in* M. jannaschii*, a modified electron transport chain reflecting the aforementioned substitution of Rnf for Ech, and an updated biomass reaction that incorporated new carbohydrate, lipid, and nucleotide composition data. Although an attempt was made to estimate the GAM purely from genomic data, the model had to retain and optimize* i*AF692's original value in order to fit experimental growth data. The biomass reaction was more systematically constructed in* i*MB745 than* i*AF692. The general components of the biomass reaction (proteins, RNA, DNA, lipids, carbohydrates, and trace components) were taken from a typical bacterial cell instead of an average methanogenic archaea cell, most likely due to the lack of experimental data. This practice is quite common when reconstructing archaeal GEMs and can have serious consequences because the biomass composition has significant influence over metabolic flux distributions throughout the network. These computed flux distributions may be biased by the use of bacterial biomass compositions rather than archaeal biomass compositions (see Tables 2 and 3 for biomass compositions from models).

**Figure 4:**
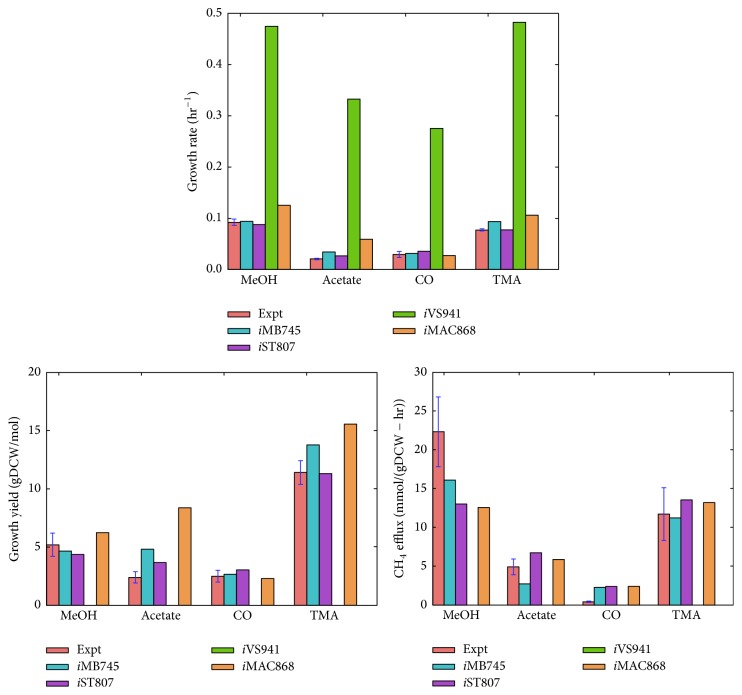
Growth characteristics of* M. acetivorans* models. The models were simulated using experimental growth substrate uptakes of MeOH:20, Acetate:7, and CO:11.6 mmol/gDCW/hr. Since experimental TMA uptake rates were not available, it was set to 6.77 mmol/gDCW/hr across all the models. This value was determined by fitting* i*ST807 to experimental growth rates on TMA.* i*VS941 gave unrealistically large growth yields and therefore the values were omitted from the growth yield plot for a clearer display of the other models' performances.* i*VS941 also did not predict any methane production under the given growth conditions. Experimental growth rates are from [30–40]. Experimental growth yields are from [40–42]. Experimental CH_4_ production rates are from [33, 42–44].

*i*VS941 was developed and published independently from* i*MB745 at the same time through homology comparison with* M. barkeri* and an automated curation procedure published by Suthers et al. [83]. The biomass reaction, which includes the GAM parameter, is directly inherited from* i*AF692, but the nucleotide compositions were modified to reflect the differences in G/C content between* M. barkeri* and* M. acetivorans*. The most recent models of the* M. acetivorans* lineage are* i*MAC868 and our own* i*ST807, both of which are independently updated metabolic models. Although the* M. smithii i*Msi385 and* M. hungatei i*Mhu428 models are indeed independent curations, we will not discuss them here because they are directly inherited from* i*MB745 and were qualified as preliminary draft models needing further revisions.* i*MAC868 was constructed to incorporate an engineered pathway that allowed for methane oxidation, essentially enabling the model to grow on methane and thus reversing the entire process of methanogenesis to produce the growth substrates that* M. acetivorans* would normally consume. Nevertheless, the model can still be used for simulations of a wild-type* M. acetivorans* and contains important updates to* i*MB745.* i*MAC868 merged the information from both* i*MB745 and* i*VS941 into a single model and corrected numerous charge and mass imbalances within the electron transport chain. 64 GPRs were also updated with the most recent* M. acetivorans* gene annotations. The biomass, GAM, and NGAM requirements remained the same as those from* i*MB745. In* i*ST807, we updated* i*MB745 by revising the methanofuran biosynthesis pathway with the most recent experimental data from* M. jannaschii* [84–87], adding 13 new reactions and 62 new genes, and revising the biomass reaction to utilize charged tRNAs instead of free amino acids. Among the new additions are reactions to enable pyrrolysine biosynthesis during methylamine growth, methyl-3-mercaptopropionate metabolism, and o-phosphoserine conversion to cysteine after aminoacylation. Being able to uptake the various media components (Wolfe medium [88]) in which* M. acetivorans* is typically grown is crucial for accurately simulating the organism's metabolism. Many of the reactions required to emulate this are either missing or turned on in* i*MB745 and* i*MAC868. Cysteine is an important media component usually added with the purpose of quenching any oxygen in the methanogen's growth environment, but no one to date has verified whether this media component is also metabolized. Since unconstraining its uptake within* i*MB745 caused erroneously high growth rates, the cysteine uptake reaction was shut off and this was inherited by* i*MAC868 along with the various missing Wolfe media uptake reactions.* i*ST807 fluxes this by incorporating uptake reactions for all the components of the Wolfe medium that have use in the metabolic network, including cysteine which is constrained to a nongrowth-limiting value that maximizes the model's agreement with the experimental growth rates shown in Figure 4.

From the methanogenic GEMs geneology, it is clear that most of the methanogenic GEMs are inherited from* i*MB745 despite the fact that* i*VS941 was independently published at the same time. This inheritance trend is most likely due to the more complete model documentations and availability of a readily testable GEM provided for* i*MB745 in contrast to* i*VS941. Given that metabolic modeling for archaea is still a developing effort, this practice of providing poorly assembled GEM files that are ill-prepared for quantitative assessment is still, unfortunately, common in the field. In order to alleviate this problem, we provide in the supplementary information (in Supplementary Material available online at https://doi.org/10.1155/2017/9763848) of this review all the currently available* M. acetivorans* models standardized to use BIGG IDs (http://bigg.ucsd.edu/data_access) and proper compartment tags such that the models can be conveniently handled within COBRApy. We also compare their growth characteristics as shown in Figure 4 to give a sense of how well these models perform with respect to each other and experimental data. We chose to focus on these models from this species because they are often used as templates for the reconstruction of many other methanogens.

*i*VS941 predicts unrealistic growth rates, growth yields, and no methane efflux, which indicates the model's deficiency. This growth characteristic assessment shows that, besides proper documentation,* i*MB745 also demonstrates better predictive ability over* i*VS941 and thus serves as a more reliable parent model for* M. acetivorans*. This is also evidenced by the predictive performances of* i*ST807 and* i*MAC868 which are updated versions of* i*MB745. Across all growth substrates and growth characteristics,* i*MAC868 predictions showed a median deviation of 36% from experimental values. In contrast,* i*ST807 demonstrated only a median deviation of 12% which is a marginal improvement over the 14% median deviation of* i*MB745. Although these statistics may seem to suggest that* i*MB745 and* i*ST807 are more reliable models overall, it is important to keep in mind that growth predictions are heavily dependent on each model's allowed uptake reactions and their respective rates. In this assessment, each model's uptake reactions were set to the defaults that were provided within their respective publications. The uptake rate for the growth substrate being tested was uniformly set to the experimental value across all the models, and all other major growth substrate uptake reactions were turned off.

## 5. Nonmethanogen GEMs

### 5.1. *Halobacterium salinarum*

While only four GEMs have been developed for only three nonmethanogenic archaea, they provided significant insight into the metabolism and growth of the organisms. A reconstruction of the halophilic archaeum* Halobacterium salinarum* R-1 capable of growing on 15 different carbon/energy sources was developed by the group of Dieter Oesterhelt [13]. During construction, a novel pentose phosphate pathway (PPP) for the generation of ribulose-5-phosphate (R5P) was predicted and later verified. It was known that different archaea used different pathways to produce R5P (e.g., nonoxidative PPP, reverse ribulose-monophosphate pathway, and oxidative PPP).* H. salinarum* was missing all or portions of these pathways. An alternate pathway using the partial Entner-Doudoroff (ED) pathway was connected to the partial oxidative branch of the PPP by a semiphosphorylated 6-phosphogluconate. This pathway thus described why the organism retained parts of the oxidative PPP and part of the ED pathway even though it is incapable of growing on sugars. During the reconstruction the authors also noted that shikimate production was incomplete and thus proposed that hexose and L-aspartate-4 semialdehyde were used, consistent with ^13^C labeling data from tryptophan degradation. Additionally, draft pathways for synthesis leucine, isoleucine, and valine could be generated in the model.

To calibrate the model, they measured the amino acid composition and content using experiments and found that protein mass constitutes 49% of the dry mass, much less than in the other archaea. Using dynamic simulations with experimentally measured uptake rates for amino acids they predicted internal fluxes from which they drew a number of conclusions. Most strikingly, only 15% of amino acid carbons ended up in biomass with the majority being used to produce energy in the TCA cycle. They found that all amino acids were simultaneously used, though arginine, aspartate, leucine, and isoleucine were taken up most quickly, even the essential amino acids methionine, lysine, isoleucine, leucine, and valine which the cells are incapable of producing. Using flux balance analysis, they found that* H. salinarum* primarily produces isoprenoid lipids using leucine (~10%) while isoleucine was primarily degraded entering the TCA as acetyl-CoA and succinyl-CoA. Valine was the only amino acid that was primarily incorporated into biomass. Because the uptake rate of amino acid far outpaced the biomass incorporation they hypothesized that degradation pathways for all amino acids exist and proposed six enzymes to facilitate some of these reactions. However, it was only later that they determined the biosynthetic pathways for aromatic amino acids which they shared in common with* M. jannaschii*; during the discovery they used the metabolic model to identify uptake rates in auxotrophs [89]. Most impressively, they predicted, and later experimentally verified, that arginine is interconverted to ornithine during its degradation and is excreted to the environment early in growth, only to be taken up later as a source of arginine. Overall, they suggested that the greedy consumption of all available amino acids results in the “blooms" observed in the wild [13] and indicates that the metabolic pathways that have evolved are such that the organism can eat as quickly as possible to outgrow competitors.

The model was later updated to include a refined description of the respiratory chain as well as phototrophic growth leading to additional insights into metabolism [14]. Several key differences in the oxidative phosphorylation pathway compared to bacteria and mitochondria were proposed. First, because complex I is missing the NADH oxidation subunits, it uses another energy carrier. Second, that halocyanin carries electrons from complex III to complex IV rather than menaquinone. Finally, that ATP synthase has a stoichiometry of 10 protons per ATP, which is much higher than in most organisms.

By fitting uptake rates of amino acids to aerobic growth experiment measurements, they identified isoleucine, leucine, and valine as the preferred energy sources, while others such as alanine, proline, and ornithine had distinct periods of different uptake rate [14]. Thus, the organism hierarchically uses metabolites to maximize growth rate. They also predicted significant overflow of alanine, acetate, and succinate. Interestingly, they identified that arginine fermentation essentially revives cell growth, after which amino acid degradation and photosynthetic growth become dominant. They found that even during anaerobic phototrophic growth, the organism breaks down amino acids to obtain energy, even though they were incapable of deriving the maximal energy from respiration. Interestingly, they could identify that the network structure of amino acid degradation could describe why alanine was produced, specifically, as an overflow pathway during serine consumption. This is in contrast to aerobic growth where serine and alanine consumption appear to coincide with one another, likely due to the fact that pyruvate can be funneled into the TCA cycle. Overall, the studies of* H. salinarum* led to the conclusion that the organism evolved its metabolic behavior to maximize growth during blooms, which can occur sporadically with many years in between [13, 14]. It was suggested that they use this as a strategy to outcompete other organisms that feed on available nutrients and build up enough of a population that they can survive long periods of starvation [14].

### 5.2. *Natronomonas pharaonis*

The metabolic network for the polyextremophile (high salt concentration and alkaline pH)* Natronomonas pharaonis* was developed [25] using the reconstruction for* H. salinarum*. The network is significantly larger, with nearly 30% more genes associated with reactions, mostly due to additional amino acid and carbon degradation pathways. As* N. pharaonis* is capable of growth on a single carbon source the reconstruction complements that for* H. salinarum* which requires a complex broth for growth. For this reason, the reconstructions could be used to investigate questions regarding the metabolic objective of halophiles that are subject to different evolutionary pressures and answer questions about optimality of energy production.

The authors measured the amino acid content to define the biomass composition and found, similar to* H. salinarum*, that it made up about 75% of organic mass [13, 25]. Perhaps the high protein content helps to compensate for the high osmolarity in which the organisms are grown. Using the model, predictions about aerobic growth were obtained; most importantly is that at very high (>7 : 3) and low (<3 : 7) acetate to oxygen consumption ratios the organism was incapable of growth. Using experiments, they identified an acetate:oxygen ratio of about 1 : 2 and an ATP maintenance cost of ~30 *μ*mol/ΔOD·mL. A wide range of maintenance energies and acetate:oxygen ratios gave near optimal growth, indicating that growth of* N. pharaonis* is robust to environment and the biological objective is to maximize growth and energy production [25]. They found that the carbon incorporation was actually quite low (~35%). Finally, using arguments about respiratory exchange ratio (e.g., the ratio between CO_2_ production and oxygen consumption) the authors were able to demonstrate that about 10% of carbon is neither incorporated in biomass nor respired, suggesting that the organism uses some form of overflow metabolism [25]. While they did not make any suggestions, there are a number of likely suspects such as succinate or pyruvate which could act as available nutrients for other organisms.

### 5.3. *Sulfolobus solfataricus*

The final nonmethanogen model developed for an archaeon is for the hyperthermoacidophile* Sulfolobus solfataricus* [26]. The model and organism are remarkable among the archaea represented here in that they grow optimally at a pH of 3.5 and temperature of 80° and consume 35 different carbon sources. The thermostability of their enzymes is of interest to bioengineers and makes the organism attractive for bioreactor design. Their unique abilities give them an edge in the hot-springs where they are found and allow them to consume a plethora of degraded organic mass. The final reconstruction consists of 706 reactions associated with 515 genes and conveys the ability to consume all 35 carbon sources. The model was calibrated with growth and nongrowth associated maintenances of 24.68 mmolATP/gDCW and 1.9 mmolATP/gDCW/hr, respectively, to match experiments. Interestingly, the GAM is the smallest of any archaea while the NGAM is moderate. Unfortunately, the model itself was not available and thus the biomass composition used in the study could not be compared with the others to identify the source of this low cost for growth (see Table 2). The authors of the study chose a phosphate/oxygen ratio of 0.5 as the final fit parameter of their model; this low value was due to the fact that the archaeon uses inefficient cytochrome complexes SoxABCD and SoxEFGHIM for respiration. Using these parameters, the model incorporates about 25% of carbon while respiring the rest.

During the model reconstruction, the authors identified the fact that* S. solfataricus* uses a reverse ribulose-monophosphate pathway (RRMP) instead of the pentose phosphate pathway. Specifically, they found that the organism was missing a transaldolase and thus they allowed accumulation of sedoheptulose 7-phosphate. They found that accumulation of sedoheptulose 7-phosphate in their simulated media accounted for ~3% of all carbon atoms and thus is a significant portion of the overall carbon available for biomass. Simulations indicated that on glucose growth about 22% of carbon flux was fed into the RRMP pathway while the rest was metabolized to pyruvate via the Entner-Doudoroff (ED) pathway to be subsequently used in TCA cycle. Flux variability analysis of the metabolic model demonstrated that both the semiphosphorylative and nonphosphorylative branches of the ED pathway were possible and indicates that further studies are required to understand the growth of the organism. Similarly, the TCA cycle showed significant variability, primarily due to the glyoxylate shunt. Finally, variability in the production of amino acids such as histidine, tryptophan, alanine, and glutamate indicates different routes of synthesis.

Because a related organism* Sulfolobus *sp. VE 6 could grow autotrophically fixing bicarbonate, the authors searched for the hydroxypropionate-hydroxybutyrate cycle. They found 11 of the 16 enzymes and performed BLAST searches to identify putative homologs of the 5 remaining enzymes. Thus, they predicted that* S. solfataricus* is able to grow autotrophically and suggested that experiments should be performed. It is important to note, however, that autotrophic growth of* S. solfataricus* had already been verified by Zillig et al. [90]. During autotrophic growth, the model predicted that the TCA cycle was little used with flux flowing from succinyl-CoA through malate to form pyruvate which could be used in gluconeogenesis. Additionally, hydrogen sulfide was fixed to provide a sulfur source, and in fact it produces energy allowing the simulated organism to grow much more quickly than on glucose; however, this is likely due to the lack of an uptake rate on H_2_S. Regardless, this gives a hint about the possibility of syntrophic interactions with sulfate reducing bacteria.

The authors went on to compare the growth of the organisms on the 35 different carbon sources. To do this they fixed the carbon uptake rate and compared biomass flux. Overall, the organism grew significantly faster when growing on glycerol and propanol and marginally better on oligosaccharides. They also grew significantly more slowly on carbon sources than on compounds that enter the TCA cycle at points other than 2-oxoglutarate. Gene deletion assays indicated that over 50% of all single gene knockouts were nonlethal and an additional ~25% allowed limited growth, suggesting* S. solfataricus* is metabolically versatile. Although* S. solfataricus* is naturally a thermophile and therefore likely possesses a large repertoire of thermostable enzymes, it has been suggested [91] that thermostability does not necessarily guarantee thermoactivity. Therefore,* S. solfataricus'* metabolic versatility is potentially advantageous in high-temperature environments, allowing the organism to circumvent the potential issues of decreased enzyme catalytic activity and higher mutation rates. Overall, these results suggest that* S. solfataricus* can preferentially consume certain carbon sources and regulate alternative pathways to support beneficial symbiotic relationships. Alternatively, one can also argue that* S. solfataricus* can take advantage of its metabolic versatility to outcompete other organisms.

## 6. Comparison of Metabolic Capabilities

Well-curated metabolic models function as comprehensive databases of the knowledge about organisms; thus, they are potentially useful tools for studying evolution and diversity of metabolism. Three properties of the metabolic models are of particular utility for comparative studies: (1) metabolic models connect gene function with metabolic function via their gene-protein-reaction rules, (2) the metabolic network is topologically defined by metabolites and the reactions that interconvert them, rather than the genes that facilitate those interconversions, and (3) metabolic networks coupled with modeling techniques allow for the identification of function redundancy/degeneracy. The first of these properties allows direct comparison of gene content based on metabolic function and the application of traditional evolutionary tools (e.g., bioinformatics and phylogenetic approaches). The second of these properties allows networks to be compared by function rather than gene content; for example, the network could be used to identify convergent evolution. The final of these properties could be used to provide insight into the selective pressures of the organism; specifically, duplicated functionality might suggest a critically important function for the organism.

**Figure 5:**
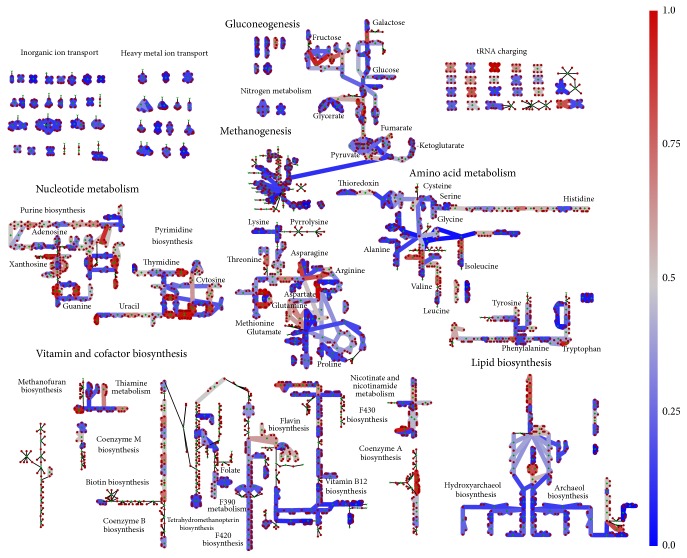
Conservation of metabolic reactions. A map showing the extent of conservation for the reactions of the* M. acetivorans* model* i*ST807 (as encoded in the gene-protein-reaction associations (GPRs) of the model). Nodes represent either a metabolite or reaction and edges indicate the dependencies between reactions and metabolites. Reactions on the blue end of the spectrum are facilitated by enzymes that are conserved in relatively few of the 221 archaea in the database while reactions in red are facilitated by highly conserved enzymes. Reactions with thin grey lines are not associated with genes. To assess conservation, the db_evaluateReactionsFromGpr.py functionality of the ITEP software [45] was used. It computes homologous genes to those in the GPRs of each reaction in* i*ST807. The ITEP function was executed with the “or” option enabled to identify whether any of the enzymes (or enzymatic subunits) annotated as facilitating the reactions were encoded in the organism.

To demonstrate the utility of metabolic models to evolutionary analyses we computed the conservation of genes facilitating metabolic reactions. An ITEP database [45] of 221 archaea, including each of the organisms in Table 1, was constructed using the default parameters. Briefly, ITEP is a software toolkit for examining microbial pan-genomes that provides functionality for constructing a BLAST database and querying protein family prediction, ortholog detection, and analysis of functional domains. Among its capabilities is assessing the GPRs of a metabolic model for each reaction and determine whether or not the homologs exist in another organism. The GPRs from the* M. acetivorans* model* i*ST807 were used as input to the db_evaluateReactionsFromGpr.py function to assess the conservation in other organisms. The “or” option to the function was used to assess whether any genes for each* M. acetivorans* reaction existed in the other archaea. Doing this for each organism, we computed the extent of conservation for each reaction (e.g., the fraction of organisms in which the reaction had conserved genes). The results can be seen in Figure 5. Given the broad comparison of many archaea against the well-curated model for* M. acetivorans*, it is not surprising that the extent of conservation is relatively low (blue) across the network. Nevertheless, it is interesting to note that the most visible conservation (red) occurs within Nucleotide metabolism, coenzyme synthesis-related reactions, and various amino acid biosynthesis reactions. This suggests that the underlying transcription and translation machinery of archaea are fairly similar.

**Figure 6:**
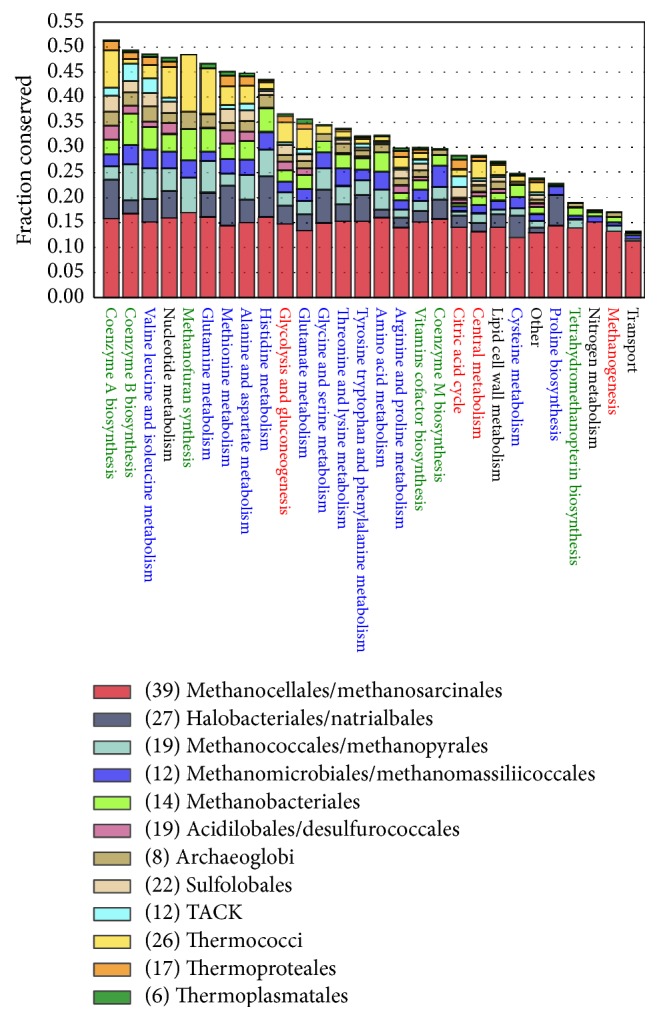
Fraction of conserved reactions. Grouping of the information shown in Figure 5 by metabolic subsystem (as annotated in* i*ST807). The overall height of each bar indicates the total fraction of reactions in the metabolic subsystem that are conserved, while the height of individual portions of each bar indicates the relative conservation of reaction in the subsystem from that organism. The 221 archaea were grouped by taxonomic order, yielding 12 distinct groups as seen in the legend. Metabolic subsystems labels are color coded: amino acid metabolism subsystems in blue, vitamin and cofactor metabolism subsystems in green, central metabolism subsystems in red, and other categories in black.

**Figure 7:**
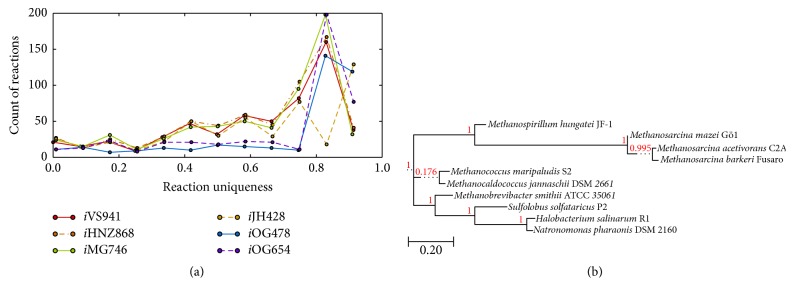
Diversity and phylogeny of metabolic models. (a) The uniqueness of the reactions in each of the metabolic models. Here, we define uniqueness to be the fraction of 221 archaea that do not have the reaction currently present in the respective model; higher uniqueness means fewer of the organisms contain the genes coding enzymes that are annotated by the GPRs of the specified models. The presence of the genes is computed using ITEP as discussed in Figure 5. (b) A phylogenetic tree computed based on similarity to the* M. acetivorans* model* i*ST807. This tree is based on the ITEP results discussed in Figure 5.

Categorizing the homologous genes by metabolic subsystem as annotated in* i*ST807 computed by ITEP lends more specific insight into conservation of metabolism in these archaea (see Figure 6). Amino acid biosynthetic pathways are generally highly conserved (labeled in blue if Figure 6). Proline and cysteine biosynthesis are notable exceptions. None of the proline biosynthesis genes annotated in* M. acetivorans* were found in type I methanogens,* H. salinarum* or* S. solfataricus*. Additionally, all genes annotated as synthesizing serine or glycine are missing in* S. solfataricus*. This could indicate either an incorrect annotation in the model or multiple proline biosynthetic pathways in metabolism. Biosynthesis of methanofuran, a cofactor in methanogenesis, is surprisingly well conserved beyond the methanogens. Notably nitrogen metabolism is the least similar among the archaea as other have previously identified [92]. Although this analysis leads to rather qualitative statements, it demonstrates how information from metabolic reconstructions can be used to compare differences between metabolism and study evolution and conservation of metabolic pathways. A similar analysis was performed with the GPRs from each of the metabolic models. The aggregate statistics from this analysis can be seen in Figure 7(a) where the count of reactions with a particular conservation level is shown. Similar to what was seen for* M. acetivorans* there appear to be mostly reactions that are highly conserved (low reaction uniqueness) or very lowly conserved (high reaction uniqueness). This is not a surprising observation as it has long been known that metabolic networks have a bowtie topology [93] and are generally scale-free networks [94]. Cross comparison between each of these conservation predictions in fine detail is beyond the scope of this review; however, we feel we have demonstrated the utility of using metabolic reconstructions as a tool to compare metabolism.

## 7. Conclusions

We have presented an overview of genome-scale metabolic models and discussed the defining metabolic features among the few GEMs available for archaea. In these discussions, we have also highlighted some much-needed improvements to model building practices in order to facilitate the development of archaeal models. By using the gene-protein-reaction associations in these archaeal GEMs, we also demonstrate the invaluable utility of these metabolic models as they can be extended beyond flux analysis to gain significant insight into evolutionary patterns among organisms. Visualizing the known archaeal metabolic models on a phylogenetic tree (see Figure 1) leads to the conclusion that model development in the community thus far has mostly focused on Euryarchaeota, leaving the Crenarchaeota largely unexplored. Although models do not yet exist for members of the Archaeoglobi, Thermoplasmata, and Thermococci classes, all other major Euryarchaeota classes have at least one representative model. This is not to underestimate the importance of further developing these Euryarchaeota models as Archaeoglobi have some of the most diverse metabolisms of any Euryarchaeota, capable of chemolithotrophy by reduction of sulfates, thiosulfates, nitrates, and heterotrophy via reduction of sulfates via organic compounds [95]. However, the paucity of GEMs for the Crenarchaeota is a major impediment for a comprehensive study of evolution and diversity in archaea. GEMs are invaluable tools to help guide the exploration and comparison of the great metabolic diversity of energy conservation in these organisms which are capable of sulfate reduction both chemolithotrophically and heterotrophically (members of the Desulfococcales), nitrate reduction (members of Thermoproteales) hydrogen oxidation, and sulfur reduction (members of the Sulfolobales) [95]. The existence of diverse energy conservation pathways will likely come with diverse electron transport chain and transport systems. Understanding these unique characteristics will be paramount in understanding growth in extreme conditions and syntrophy among microorganisms as well as for engineering communities for biotechnology applications.

## Supplementary Material

The supplementary data includes modified versions of the four previously published models for Methanosarcina acetivorans where reaction and metabolite identifiers have been standardized so as to allow direct comparison. These models were used for the comparative study of M. acetivorans presented in the review. Models are presented in the SBML format which is compatible with COBRAPy and COBRA Toolbox software packages. Models were originally published as: 1) iVS941 in Kumar et al. 2011, 2) iMB745 in Benedict et al. 2012, 3) iMAC868 in Nazem-Bokaee et al. 2016, and 4) iST807 in Peterson et al. 2016.

## Figures and Tables

**Table 1:** *Model Statistics*. The elementary units of genome-scale metabolic models are genes, metabolites, and reactions. These models consist of (1) a metabolic network that describes the connections between metabolites through reactions (often organized by metabolic subsystem), (2) gene-protein-reaction rules that map the gene dependencies of reactions, and (3) connection to their environment through exchange transport reactions.

Organism	Model	Genes	Metabolites	Reactions	Transport	Subsystems	Citation
*H. salinarum*	*i*OG490	490	557	711	111	NA	[13]
	*i*OG478	478	545	664	97	NA	[14]

*M. acetivorans*	*i*VS941	941	708	705	71	60	[15]
	*i*MB745	745	715	818	69	30	[16]
	*i*MAC868	868	707	839	91	31	[17]
	*i*ST807	807	733	759	70	30	[18]

*M. barkeri*	*i*AF692	692	558	619	88	8	[19]
	*i*MG746	746	718	815	74	31	[20]

*M. hungatei*	*i*Mhu428	428	639	721	41	29	[21]

*M. jannaschii*	*i*TS436	436	510	609	1	113	[22]

*M. maripaludis*	*i*MM518	518	605	570	49	117	[23]

*M. mazei*	*i*SS85	NA	74	85	5	NA	[6]

*M. smithii*	*i*Msi385	385	582	525	35	NA	[24]

*N. pharaonis*	*i*OG654	654	597	683	88	NA	[25]

*S. solfataricus*	*i*TU515	515	705	718	58	65	[26]

**Table 2:** *Cellular Molar Fractions*. The composition of each Archaea's biomass organized by major categories.

Molecule	*i*OG490/*i*OG478	*i*VS941	*i*MB745/*i*MAC868^a^/*i*ST807	*i*AF692	*i*MG746	*i*Mhu428	*i*MM518	*i*Msi385	*i*OG654	*i*TU515^b^
Amino acids	0.894	0.869	0.889	0.858	0.852	0.889	0.662	0.904	0.908	NA
DNA	0.003	0.018	0.013	0.016	0.008	0.013	0.013	0.016	0.012	NA
RNA	0.086	0.090	0.076	0.102	0.100	0.076	0.080	0.077	0.066	NA
Lipids	0.015	0.009	0.008	0.009	0.009	0.008	0.277	0.002	0.013	NA
Carbohydrates	NA	0.002	0.009	0.002	0.008	0.009	0.0004	NA	NA	NA
Soluble pool	0.001	0.013	0.006	0.013	0.016	0.006	0.0081	NA	0.001	NA

^a^The  *i*MAC868 model adopted the *i*MB745 biomass expression verbatim. ^b^The  *S. solfataricus* paper does not detail the biomass components and the model was not available to query. “Soluble pool” includes various vitamins, cofactors, and trace metals.

**Table 3:** *Biomass Compositions and Energy Requirements*. Molar composition of each Archaea's biomass for nuclei and amino acids. Additionally, energy requirements for growth and persistence.

Molecule^a^	*i*OG490/*i*OG478	*i*VS941	*i*MB745/*i*MAC868^b^/*i*ST807	*i*AF692	*i*MG746	*i*JH428	*i*MM518	*i*Msi385	*i*OG654^c^	*i*TU515^d^
dAMP	0.0025	0.0360	0.0234	0.0331	0.0327	0.0232	0.0370	0.0331	0.027	NA
dCMP	0.0049	0.0234	0.0175	0.0215	0.0212	0.0176	0.0196	0.0215	0.016	NA
dGMP	0.0025	0.0234	0.0175	0.0215	0.0212	0.0176	0.0173	0.0215	0.016	NA
dTMP	0.0051	0.0360	0.0237	0.0331	0.0327	0.0236	0.0376	0.0231	0.027	NA
AMP	0.0618	0.0012	0.143	0.1846	0.1782	0.1152	0.1795	0.2222	0.155	NA
CMP	0.131	0.1637	0.115	0.1379	0.1361	0.1008	0.1780	0.1379	0.086	NA
GMP	0.131	0.2637	0.101	0.2222	0.2193	0.1200	0.1609	0.2222	0.086	NA
UMP	0.0619	0.1767	0.120	0.1489	0.1469	0.1440	0.1877	0.1489	0.155	NA

Ala	0.345 ± 0.08	0.5621	0.388	0.5621	0.5546	0.3906	0.5853	0.5621	0.557 ± 0.005	NA
Arg	0.211 ± 0.044	0.3237	0.253	0.3237	0.3194	0.2520	0.2805	0.3237	0.378 ± 0.022	NA
Asp	0.431 ± 0.094	0.2638	0.301	0.2638	0.2603	0.3024	0.2805	0.2638	0.913 ± 0.080	NA
Asn	0.099	0.2638	0.253	0.2638	0.2603	0.2520	0.2805	0.2638	NA	NA
Cys	0.033	0.1002	0.07	0.1002	0.0989	0.0693	0.0915	0.1002	0.056	NA
Glu	0.72 ± 0.22	0.288	0.450	0.2880	0.2842	0.4473	0.3170	0.288	1.074 ± 0.135	NA
Gln	0.125	0.288	0.143	0.2880	0.2842	0.1449	0.3170	0.288	NA	NA
Gly	0.29 ± 0.053	0.6704	0.408	0.6704	0.6615	0.4095	0.4695	0.6704	0.561 ± 0.049	NA
His	0.133 ± 0.026	0.1037	0.094	0.1037	0.1023	0.0945	0.0976	0.25	0.104 ± 0.009	NA
Ile	0.137 ± 0.031	0.3179	0.415	0.3179	0.3137	0.4158	0.2683	0.3179	0.272 ± 0.012	NA
Leu	0.251 ± 0.052	0.493	0.534	0.4930	0.4865	0.5355	0.5182	0.493	0.471 ± 0.029	NA
Lys	0.115 ± 0.023	0.3755	0.370	0.3755	0.3705	0.3717	0.3292	0.3755	0.219 ± 0.017	NA
Met	0.05	0.1682	0.132	0.1682	0.1660	0.1323	0.1341	0.1682	0.028 ± 0.012	NA
Phe	0.111 ± 0.022	0.2027	0.251	0.2027	0.2000	0.2520	0.1829	0.2027	0.242 ± 0.018	NA
Pro	0.111 ± 0.022	0.2419	0.225	0.2419	0.2387	0.2268	0.2073	0.2419	0.358 ± 0.034	NA
Pyl	NA	NA	NA/NA/0.0808	NA	NA	NA	NA	NA	NA	NA
Ser	0.22 ± 0.053	0.2361	0.390	0.2361	0.2330	0.3906	0.2683	0.2361	0.332 ± 0.016	NA
Thr	0.181 ± 0.036	0.2776	0.307	0.2776	0.2739	0.3087	0.2927	0.2776	0.415 ± 0.011	NA
Trp	0.052	0.0622	0.060	0.0622	0.0614	0.0567	0.0061	0.0622	0.076	NA
Tyr	0.048 ± 0.023	0.1509	0.210	0.1509	0.1489	0.2079	0.1585	0.1509	0.166 ± 0.028	NA
Val	0.25 ± 0.057	0.4631	0.387	0.4631	0.4570	0.3906	0.4085	0.4631	0.480 ± 0.061	NA

GAM^e^	NA	70.0	65.0	70.0	65.0	47.0	29.8	50	30 ± 4^c^	24.86
NGAM^f^	2.0	1.75	2.5	1.75	2.0	0.6	0.4	NA	2.0^c^	1.9

^a^Unless otherwise noted the units of the biomass coefficients are in units of mmol/gDCW. ^b^The  *i*MAC868 model adopted the *i*MB745 biomass expression verbatim. ^c^The model was formulated in units of mol/OD-L. ^d^The  *S. solfataricus* paper does not detail the biomass components and the model was not available to query. ^e^Growth Associated Maintenance (GAM) has units of mmol ATP/gDCW. ^f^Nongrowth Associated Maintenance (NGAM) has units of mmol ATP/gDCW/hr.
